# Update on Transanal NOTES for Rectal Cancer: Transitioning to Human Trials

**DOI:** 10.1155/2012/287613

**Published:** 2012-05-20

**Authors:** Dana A. Telem, David L. Berger, Liliana G. Bordeianou, David W. Rattner, Patricia Sylla

**Affiliations:** Division of Gastrointestinal Surgery, Massachusetts General Hospital, 15 Parkman Street, Wang 460, Boston, MA 02114, USA

## Abstract

The feasibility of natural orifice translumenal endoscopic surgery (NOTES) resection for rectal cancer has been demonstrated in both survival swine and fresh human cadaveric models. In preparation for transitioning to human application, our group has performed transanal NOTES rectal resection in a large series of human cadavers. This experience both solidified the feasibility of resection and allowed optimization of technique prior to clinical application. Improvement in specimen length and operative time was demonstrated with increased experience and newer platforms. This extensive laboratory experience has paved the way for successful clinical translation resulting in an ongoing clinical trial. To date, based on published reports, 4 human subjects have undergone successful hybrid transanal NOTES resection of rectal cancer. While promising, instrument limitations continue to hinder a pure transanal approach. Careful patient selection and continued development of new endoscopic and flexible-tip instruments are imperative prior to pure NOTES clinical application.

## 1. Introduction

 Just as laparoscopy resulted in a major paradigm shift in the field of gastrointestinal surgery, NOTES has the potential to be equally as ground breaking and likely represents the next step in the evolution of minimally invasive surgery [1]. Proposed advantages of NOTES include faster recovery time, shorter hospital stays, improved pain control, and avoidance of potential abdominal wall complications including wound infection and hernia [2]. The range of operations under investigation is rapidly increasing. Currently, transvaginal, transgastric, transesophageal, and transanal approaches have been described. The international and national experience now counts several thousand cases of successfully performed hybrid transvaginal NOTES procedures including but not limited to cholecystectomy, nephrectomy, and vertical sleeve gastrectomy [3–9]. Progress however, continues to be hampered by instrument limitations as well as safety concerns regarding NOTES translumenal access, particularly regarding access closure.

Transanal access for colon resection has been proven safe and feasible in both swine and fresh human cadaveric models [10, 11]. The advantages of transanal access for colorectal resection are multiple. First, the availability of well-established platforms such as transanal endoscopic microsurgery (TEM) to gain access to the peritoneal cavity facilitates performance of endorectal and transrectal procedures [12]. Second, creation of the enterotomy though the organ to be resected rather than an otherwise healthy organ obviates concerns regarding safe, reproducible closure associated with other NOTES access points. In 2007, Whiteford et al. described the first transanal NOTES radical sigmoidectomy in human cadavers [13]. Although colon and mesenteric dissection could be technically achieved with use of the TEM platform, difficulties were encountered with mobilization of adequate specimen length secondary to instrument inability to overcome anatomic constraints. While instrument limitations continue to be a barrier to pure application of transanal NOTES resection, this approach has since been optimized in both a swine and fresh human cadaveric model. Based on this work, human clinical trials are currently underway worldwide [14–16].

The aim of this paper is to provide a review of our experience with transanal NOTES colorectal resection as well as an update on the current status of human clinical trials worldwide.

## 2. Technique Development

**Figure 1:**
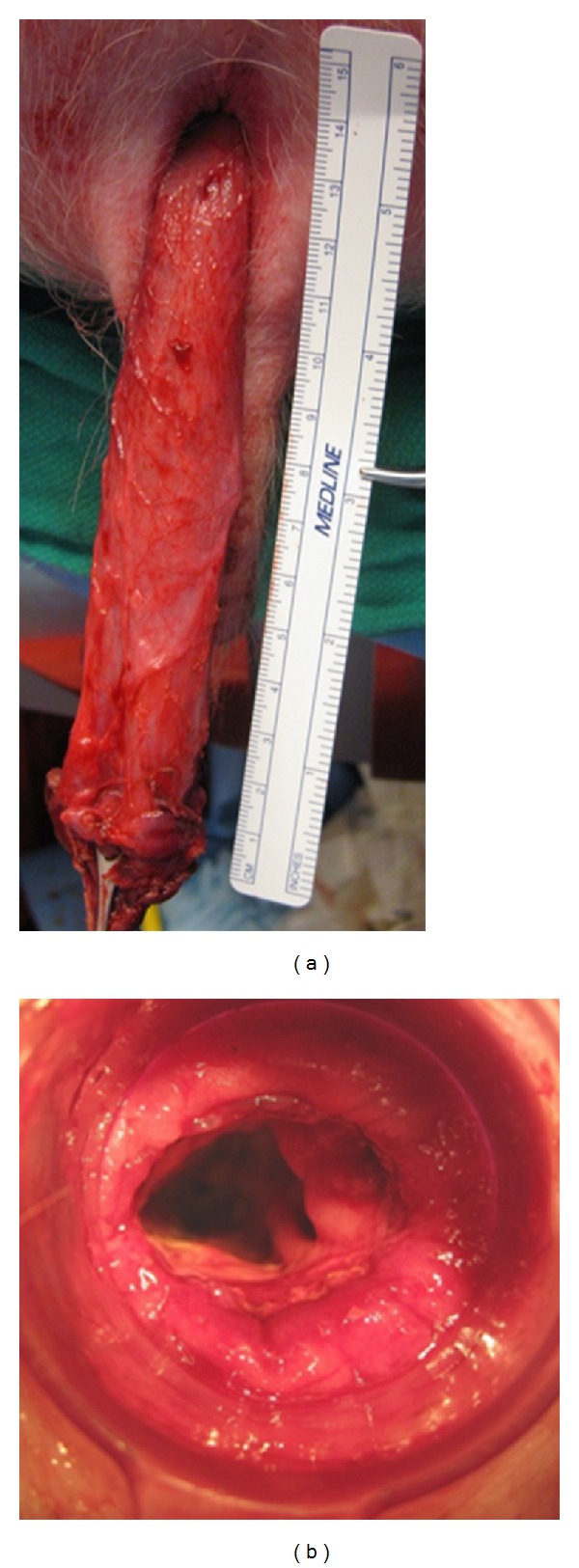
(a) Tranasanal extraction of specimen following completely NOTES in a swine survival model. (b) Intact stapled coloanal anastomosis following specimen transection.

To determine the feasibility of transanal NOTES rectosigmoid resection, a pilot study using a nonsurvival porcine model was performed [11]. Rectosigmoid resection using the TEM platform was replicated in this model. A purse-string suture was placed in the distal rectum to prevent fecal outflow and contamination. Following this, full-thickness incision of the rectal wall was performed. Upon entry into the presacral space, en-bloc resection of the rectosigmoid colon and its mesentery could be performed endoscopically. Once the peritoneal reflection was reached, the peritoneal cavity was entered and dissection of the sigmoid colon continued proximally until anatomic and instrument limitations were encountered. The colon was then pulled out through the anus, transected and a stapled colorectal anastomosis performed. Figures 1(a) and 1(b).

From this nonsurvival model, several key limitations were identified and addressed. First, the sharp angle of the sacral promontory and narrow swine pelvis hindered proximal dissection. In an attempt to overcome these anatomic limitations, a combined transgastric and transanal approach was attempted. While prolonging operative time, dual transanal and transgastric approach improved visualization, retraction, and ultimately mobilization of the proximal colon yielding additional specimen length. The addition of transgastric endoscopic access resulted in an average gain of 5.8 cm in colon length [11]. Other anatomic constraints included the flaccid swine bladder which obscures the rectosigmoid, spiral colon configuration, and lack of a true splenic flexure making proximal colonic mobilization more challenging. To overcome these anatomic challenges, exposure was improved by positioning animals in the Trendelenburg and right lateral decubitus position.

A second impediment centered on technical optimization of the colorectal anastomosis. A stapled colorectal anastomosis was performed in all animals in this series. Following anastomotic inspection the staple line was noted to be incomplete in 2 out of 9 (22%) animals. A small posterior anastomotic defect was identified in each case and believed to be secondary to an incomplete purse-string suture on the open distal rectum. This discovery led to technique modification. A transanal purse string was placed under direct vision using anal retractors, rather than through the proctoscope, with improved results [11]. 

Despite technical and anatomic limitations, all resected specimens were intact with respect to colon wall and attached mesentery. Given the promising results regarding the feasibility of this approach, the next step involved determining safety of application. A two-week survival study using 20 swines was initiated [10]. This study compared outcomes of pure transanal endoscopic resection versus combined transanal and transgastric rectosigmoid resection as described in the pilot study. All procedures were performed successfully without transabdominal assistance and all specimens were grossly intact with respect to integrity of colonic wall and attached mesentery. The use of transgastric assistance again demonstrated a significant increase in the length of specimen able to be mobilized and resected. No mortalities occurred in either group. Two morbidities, one intraabdominal abscess and one abdominal wall hematoma, occurred in the dual transgastric and transanal group identified at necropsy. Experimental evidence from both the nonsurvival and survival swine studies demonstrated both the feasibility and safety of transanal NOTES rectosigmoid resection using TEM with or without transgastric endoscopic assistance. This work served as the foundation for transitioning to human application.

### 2.1. Technique Optimization and Transitioning to Clinical Application

In preparation for human application, fresh human cadaveric models were utilized to optimize this technique. The purpose of this model was to both determine the technical and oncologic feasibility of this technique and eventually optimize this procedure for human clinical trials. Since initiation of this protocol, transanal NOTES rectosigmoid resection has been successfully performed in 32 fresh human cadavers [17]. NOTES transanal endoscopic rectosigmoid resection was performed using transanal dissection alone (*n* = 19), with transgastric endoscopic assistance (*n* = 5) or with laparoscopic assistance (*n* = 8). Of the 19 cadaveric operations performed via a pure transanal approach, 2 were performed using laparoscopic and TEO instruments through the TEO platform, 8 using endoscopic assistance with a gastroscope (Pentax) inserted through the TEO platform, and 9 utilized endoscopic assistance through a novel rigid endoscopic platform inserted through the TEO platform (ISSA, Storz). The purpose of this novel platform was to provide additional rigidity to the gastroscope.

### 2.2. Technique

**Figure 2:**
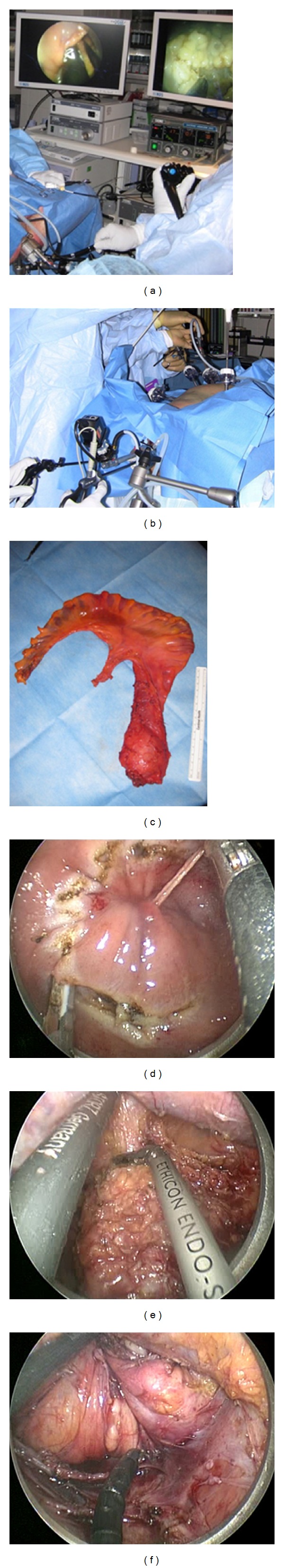
(a) Set up for pure NOTES transanal rectosigmoid resection via TEM using standard instruments and endoscopic tools in cadavers using a colonoscope for visualization. (b) Set up for transanal NOTES rectosigmoid resection with laparoscopic assistance in cadavers. (c) Transanal circumferential and full-thickness rectal dissection through the TEM platform, starting just below the purse-string suture, in a female patient with an upper rectal cancer. (d) Transanal posterior mesorectal dissection using laparoscopic instruments through the TEM platform in a female patient. (e) Transanal mobilization of the anterior rectal wall and peritoneal entry through the TEM platform in a female patient. (f) Intact rectosigmoid specimen and mesorectum following transanal NOTES procedures.

As in swine, the rectum was occluded transanally with a 2-0 vicryl purse-string suture approximately 3-4 cm from the anal verge, above the sphincter complex. The 7.5 cm TEO proctoscope (Storz, Tuttlingen, Germany) was then inserted transanally and sealed with a faceplate. CO_2_ was then insufflated (Figures 2(a) and 2(b)). Circumferential dissection of the rectum was initiated above the anal sphincter complex using electrocautery and TEO dissecting instruments (Figure 2(c)). Low pressure CO_2_ insufflation (9 mm Hg) was used to facilitate dissection. Posterior entry into the presacral space was facilitated by CO_2_ insufflation and flexible-tip instruments. The mesorectum was mobilized sharply, with or without electrocautery or a bipolar device (Autosonix ultrashears, Covidien, Norwalk, CT), and mesorectal dissection proceeded cephalad along the avascular presacral plane (Figure 2(d)). This plane of dissection was extended medially, laterally, and anteriorly to achieve circumferential rectal mobilization and TME. The shorter proctoscope was replaced with the 15 cm proctoscope to improve exposure. The peritoneal reflection was visualized and divided anteriorly after carefully mobilizing the vagina or prostate from the anterior rectal wall, and the peritoneal cavity was entered (Figure 2(e)). The peritoneal attachments of the rectosigmoid were divided using electrocautery and a bipolar device (Autosonix). Proximal dissection was continued either via transanal endoscopic dissection alone or with transgastric endoscopic or laparoscopic assistance. The inferior mesenteric pedicle was taken in all cadavers using a bipolar device or a linear endoscopic stapler (EndoGIA, Covidien) inserted transanally through the TEO platform.

In cadavers undergoing sole transanal rectosigmoid resection, dissection into the peritoneal cavity was extended as cephalad as possible using TEO and laparoscopic instruments, with or without transanal endoscopic assistance using a gastroscope (Pentax Medocal Incl, Montvale, NJ, USA). When dissection could not be extended any further, the proctoscope was removed, and the specimen was exteriorized in preparation for specimen extraction.

Transgastric assistance, when utilized, was performed as previously described [10]. In brief, following maximal transanal rectosigmoid mobilization, peroral transgastric peritoneal access was obtained using a 12.8 mm colonoscope (Pentax). A 4 mm gastrostomy was then made using a needle knife (Cook Medical Inc., Winsont-Salem, NC, USA) and dilated. Once access was established, the colonoscope was advanced into the peritoneal cavity. In 2 cases, transgastric access and dissection were performed using a novel endoscopic platform (Anubiscope, Storz). The lateral peritoneal attachments of the rectosigmoid, sigmoid, and descending colon was then divided using the needle knife. Transanal and transgastric mobilization were combined until no further mobilization could be safely achieved. For operations performed with laparoscopic assistance, 1–3 abdominal trocars were inserted to improve visualization and/or facilitate colon retraction. This permitted more proximal dissection of the rectosigmoid junction.

Regardless of operative approach, once the rectosigmoid specimen had been fully mobilized, it was exteriorized transanally, measured and subsequently transected (Figure 2(f)). A Lone Star retractor (Cooper Surgical, Trumbull, CT, USA) was then positioned and a handsewn coloanal anastomosis performed between the proximal sigmoid colon and distal anorectal cuff as previously described.

### 2.3. Technical Feasibility and Optimization

In this series of 32 fresh human cadavers, 21 were male and 11 female with mean BMI of 24 kg/m^2^. Mean operative time was 5.1 hours and mean specimen length 53 cm (range 15 to 91.5 cm). A significant improvement in both specimen length and operative time was demonstrated with increased experience [17]. In addition, comparison by operative approach demonstrated significantly improved specimen length with addition of laparoscopic assistance. Cases that employed a hybrid transgastric and transanal approach initially resulted in increased specimen length; however, this became less pronounced with increasing experience in transanal dissection alone. In 8 (25%) cadavers, an enteric perforation was identified in the sigmoid (*n* = 2), rectum (*n* = 3), or proximal colon (*n* = 2). Factors associated with complication included obesity, poor cadaver quality, pelvic adhesions, and a redundant sigmoid colon. In addition, all enteric perforations occurred in cadavers undergoing pure NOTES rectosigmoid resection during attempted mobilization of the proximal descending colon. Limitations in dissecting instruments, current platforms, and proximal visualization are likely responsible for the rate of enteric perforation. While the feasibility of pure NOTES colorectal resection could be replicated in fresh human male and female cadavers, the complication rate highlights that clinical application is not yet possible and a hybrid laparoscopic approach is essential. In addition to serving as an experimental platform, this model also enabled standardization of a hybrid laparoscopic procedure prior to clinical trials. It allowed for the capability of trouble shooting and overcoming the procedural learning curve prior to human application.

### 2.4. Oncologic Feasibility

Another question that needed to be addressed prior to transitioning to human trials pertained to the adequacy of oncologic resection. Both cadaveric work done by our group as well as the one by Whiteford et al. [13] illustrate that this operation is oncologically appropriate. As total mesorectal excision (TME) remains the gold standard in the treatment of rectal cancer, we evaluated oncologic adequacy in our cadaveric model by specimen assessment following procedure. In our series of 32 cadavers, the mesorectum was intact in 100% of specimens following TME. The capability of performing an adequate oncologic operation was corroborated in 2011 by Rieder et al. [18]. This paper randomized male cadavers to either laparoscopic or transanal sigmoid resection for a lesion simulated at 25 cm. Lymph node yield as well as adequate resection margins were evaluated. This study demonstrated similar lymph node yield following transanal rectosigmoidectomy when compared to the laparoscopic approach. Given the distance of the simulated lesion however, laparoscopic assistance was necessary in the transanal group to achieve adequate proximal resection margin. Nonetheless, results from this study support the feasibility of this technique as an adequate oncologic procedure.

## 3. Clinical Trials

Success in animal and cadaveric models has led to worldwide human clinical trials [14–16]. In 2010, our group reported the first hybrid NOTES transanal total mesorectal excision (TME) in a 76-year-old female with a T2N1 rectal cancer treated preoperatively with neoadjuvant therapy [16]. Visualization and assistance during the procedure were aided with a transabdominal 5 mm port that later became the stoma site and 2 mm needle ports of which one was used as a drain site. The TME was performed entirely transanally through the TEO platform (Storz, Tuttlingen, Germany) with mobilization of the splenic flexure and proximal intra-abdominal colon performed laparoscopically. The specimen was then transected transanally and a handsewn coloanal anastomosis with diverting loop ileostomy was performed. The operative time was 4 hours and 30 minutes. The patient did well postoperatively and was discharged home on postoperative day four. The final pathology demonstrated a ypT1N0 tumor with intact mesorectum that included 23 negative lymph nodes and negative proximal, distal and radial margins. The patient later underwent ileostomy reversal with good function and has remained free of disease.

 Since this report, 3 additional cases have been reported in the literature. Zorron et al. published a series of 2 patients who underwent successful hybrid NOTES TME for rectal cancer [14]. In this series, mesorectal dissection is described with both an endoscope and with a transrectal rigid single port device. The first case was that of a 54-year-old male who presented with an adenocarcinoma 8 cm from the rectal verge causing 90% stenosis of the lumen. Secondary to the obstructing nature of his tumor, the patient did not undergo neoadjuvant therapy. Hybrid transcolonic NOTES TME was performed using a colonoscope. Following identification of the anal verge, a 2.5 cm posterior incision was performed in the planned line of rectal resection. The colonoscope was then inserted directly into the perirectal retroperitoneal space and dissection was performed by directing the endoscope via CO_2_ insufflation through a working channel. Once dissection reached the level of the peritoneal cavity, pneumoretroperitoneum was lost and dissection was then facilitated by laparoscopic assistance via 3 transabdominal trocars. Once dissection was complete, the specimen was removed transanally and a stapled anastomosis and right transverse diverting colostomy were performed. Operative time was 350 minutes. Both the intra- and postoperative courses were uncomplicated and the patient was discharged home on postoperative day 6. Pathology revealed an intact mesorectum with 3 out of 12 retrieved lymph nodes positive for tumor (pT3N1). Margins were free of tumor.

 The second patient reported in this series was a 73-year-old female with a diagnosis of rectal adenocarcinoma 6 cm from the anal verge who underwent neoadjuvant therapy. In contrast to the first patient, this patient underwent a hybrid NOTES TME using a transanally inserted rigid, single port device. The single port access device has 3 channels for instrumentation, with 2 additional channels for CO_2_ insufflation. Using a 10 mm 45-degree laparoscopic camera, in lieu of a flexible colonoscope, the TME dissection was then carried out transanally with laparoscopic assistance as previously described. Operative time was 360 minutes. This patient also recovered uneventfully and was discharged home on postoperative day 6. Pathology revealed tumor-free margins and intact mesorectum with 2 of 11 lymph nodes positive for tumor (pT3N1).

 The third case was reported by Tuech et al. in 2011 [15]. This report describes a 45-year-old woman with a reported T1sm3 rectal adenocarcinoma 3 cm above the dentate line. For this procedure a single port access device, endorec trocar (Aspide, France), was also used. This trocar consists of a rigid port with 40 mm outer diameter, three 5 mm, and one 10 mm access channel and an air inlet tube through which CO_2_ can be inflated. The extraperitoneal rectum was completely mobilized using this device. Once the lateral rectal attachments were divided, the rectovaginal peritoneal reflection was identified and perforated to gain access to the abdominal cavity. A second endorec trocar (Aspide, France) was then placed through the proposed ileostomy site and laparoscopic assistance with proximal colonic mobilization ensued. The procedure was performed successfully without complication. Operative time was 5 hours. The patient did well postoperatively without complication. Pathology revealed a pT1sm3N0 tumor. Fifteen lymph nodes were retrieved with the specimen.

While the principles of NOTES transanal rectal cancer resection remain the same, the methodology, particularly with respect to transanal dissection, varies between clinical trials. The consensus is that the majority of the rectal and mesorectal dissection can be achieved transanally while laparoscopic assistance is needed for proximal colon mobilization and tissue retraction. It is the preference of our group at this time to use the rigid TEO platform for transanal endoscopic rectal dissection rather than a flexible single port device. The TEO platform comes in 2 lengths, provides rigid stabilization for instrument manipulation, and is an established cost effective, reusable platform readily available at our institution. Nonetheless, the published reports thus far demonstrate that adequate hybrid NOTES TME can be achieved using flexible or rigid platforms and highlight the importance of continued work and development in this field.

 As part of our effort to further this work, we are currently enrolling patients into an ongoing United States based Institutional Review Board (IRB) approved prospective clinical trial [19]. Patients selected for this approach include those with biopsy proven resectable adenocarcinoma of rectum located 4–12 cm from anal verge who are otherwise eligible to undergo standard open or laparoscopic low anterior resection with temporary diverting stoma. Tumors must be preoperatively staged as node negative, T1 (high risk features), T2 or T3 based on pelvic MRI with no evidence of metastasis on staging CT scans. For preoperatively staged T3N0 tumors, patients must have completed full-course neoadjuvant treatment. Procedures are performed following the same steps as described in cadavers, using an abdominal and perineal team working simultaneously. Transanal dissection is performed via the TEM platform with laparoscopic assistance through 1–4 abdominal trocars. The right lower quadrant trocar is later used as the ileostomy site. Following transanal specimen retrieval, a handsewn coloanal anastomosis with diverting ileostomy is performed. For this protocol, a diverting ileostomy is standard given performance of a low-lying anastomosis in patients who likely will require either neoadjuvant or adjuvant chemoradiation.

## 4. Conclusion

Transanal NOTES rectosigmoid resection is feasible and safe as demonstrated in both a swine and fresh human cadaveric model. Clinical application has been promising, with several hybrid laparoscopic and transanal procedures for rectal cancers published to date. While encouraging, instrument limitations continue to hinder a pure transanal approach. Continued development of new flexible endoscopic platforms and flexible-tip instruments are imperative prior to pure NOTES clinical application in humans. In addition, the success of clinical application will ultimately rely on careful patient selection and strict adherence to oncologic principles of resection with all planned procedures done in the setting of IRB-approved clinical trials.
